# Brain imaging findings in Parkinson disease with Pisa syndrome: A case report

**DOI:** 10.1097/MD.0000000000024631

**Published:** 2021-02-12

**Authors:** Cheng-Jui Jamie Hung, Su-Chen Wang, Yuan-Yang Cheng, Shin-Tsu Chang

**Affiliations:** a School of Medicine, Chung Shan Medical University, Taichung; b Department of Long Term Care and Management, Chung Hwa University of Medical Technology, Tainan; c Department of Physical Medicine and Rehabilitation; d Center for Geriatrics and Gerontology, Taichung Veterans General Hospital, Taichung; e School of Medicine, National Yang-Ming University; f Department of Physical Medicine and Rehabilitation, Tri-Service General Hospital, School of Medicine, National Defense Medical Center, Taipei; g Department of Medicine, Chung Shan Medical University, Taichung, Taiwan.

**Keywords:** basal ganglion, ethyl cysteinate dimer, Parkinson disease, Pisa syndrome, single-photon emission computed tomography

## Abstract

**Rationale:**

The Pisa syndrome (PS) is defined as a kind of reversible postural deformity which causes a lateral trunk flexion of 10 degrees or more. A prevalence of approximately 7.4% to 10.3% of patients with Parkinson disease (PD) also have PS. Though unbalanced function of the basal ganglia network and impaired visual-spatial functions including parietal cortices in PS is known, the pathophysiology of PS remains to be unclear.

**Patient concerns:**

A 67-year-old male patient with PD visited our Rehabilitation outpatient department because of his trunk which involuntarily deviated to the left side when he stood up.

**Diagnoses:**

Based on the history, physical examination, X-ray images, Tc-99m brain TRODAT-1 single-photon emission computed tomography (SPECT), and regional cerebral perfusion Tc-99m ethyl cysteinate dimer SPECT, the patient was diagnosed with PD with PS.

**Interventions:**

The patient refused our recommendation of admission for pharmaceutical treatment due to personal reasons and was only willing to accept physical training programs at our outpatient department.

**Outcomes:**

We arranged functional neuroimaging of the brain to survey possible neurologic deficits. The patient's images of ethyl cysteinate dimer SPECT and TRODAT SPECT showed abnormalities, including hypoperfusion and diminished dopamine transporter uptake, in the areas of the basal ganglia network and other brain regions.

**Lessons:**

Based on previous literature and the imaging of our patient, we hypothesize that PS results from unbalanced function of the basal ganglia network and impaired visual-spatial functions of bilateral parietal cortices.

## 1 Introduction

The Pisa syndrome (PS) is defined as a reversible postural deformity which leads to a lateral trunk flexion of 10 degrees or more and can be fully mitigated by passive mobilization or in the supine position.^[1]^ Among various clinical reasons resulting in the PS, Parkinson disease (PD) is one of them. While postural deformities are common in patients with PD, the PS influences patients with PD with a prevalence of 7.4% to 10.3%.^[2]^

Despite the exact pathogenesis of PS being undefined, the dystonic activity and impaired sensorimotor integration observed in PD patients with PS may result from defects in their central nervous system.^[1,3]^ The presentation of unilateral trunk flexion in the PS implies its mechanism possibly involves interruptions from neural pathways or nuclei in motor control, such as the basal ganglia. Although brain circulation scans were uncommonly arranged in PD, there are some values in these images. We herein report a case of an elderly male patient of PD with PS, whose brain circulation images showed abnormalities in the areas of the basal ganglia network and other brain regions.

## 2 Case report

The patient is a 67-year-old male with past history of severe osteoarthritis of right hip osteoarthritis s/p total hip replacement, and spondylolisthesis of L3/4/5 with L4/5 spinal stenosis s/p transforaminal lumbar interbody fusion of L3-L5 on the 5th of July 2019, and later screw removal due to loosening and cage migration.

The patient was diagnosed with PD approximately 15 years ago with initial presentation of resting tremor in his right hand and weakness in his left lower limb. Bradykinesia developed 5 years after the onset of PD. Steppage gait has also been noted for some years but without clear time of onset. Besides, the patient also noticed his trunk flexing to the left side, and the symptom of trunk lateral flexion exacerbated in the recent 2 years. On February 25, 2020, the patient visited the outpatient department (OPD) of our Department of Rehabilitation, in which he complained his trunk involuntarily deviated towards the left side while standing. Meanwhile, he also experienced numbness and pain in both lower limbs for more than 5 months and difficulties in raising his left lower limb.

**Figure 1:**
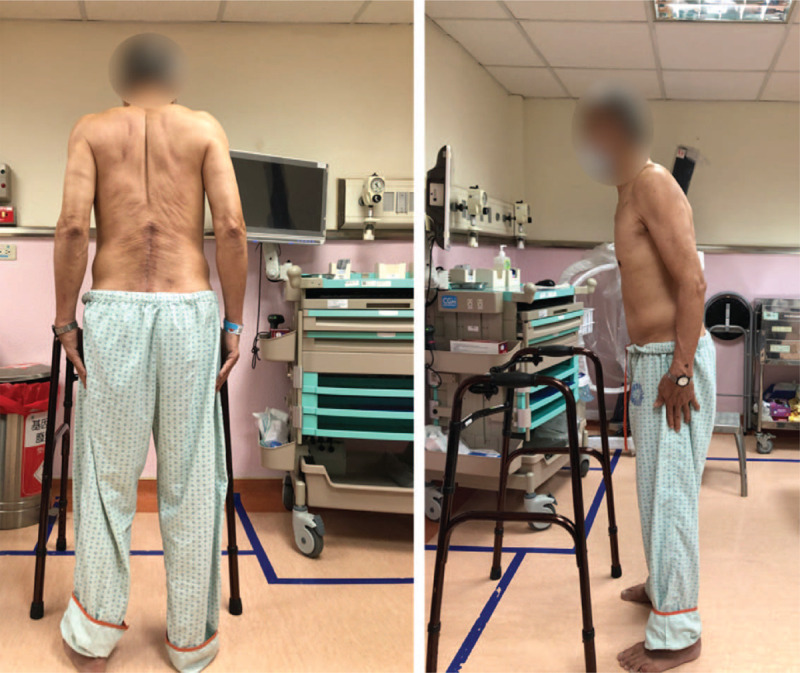
Pictures of the patient taken in the hospital. These 2 pictures show the patient's trunk slightly leans to the left side and the patient is not able to stand straight without the assistive device.

**Figure 2:**
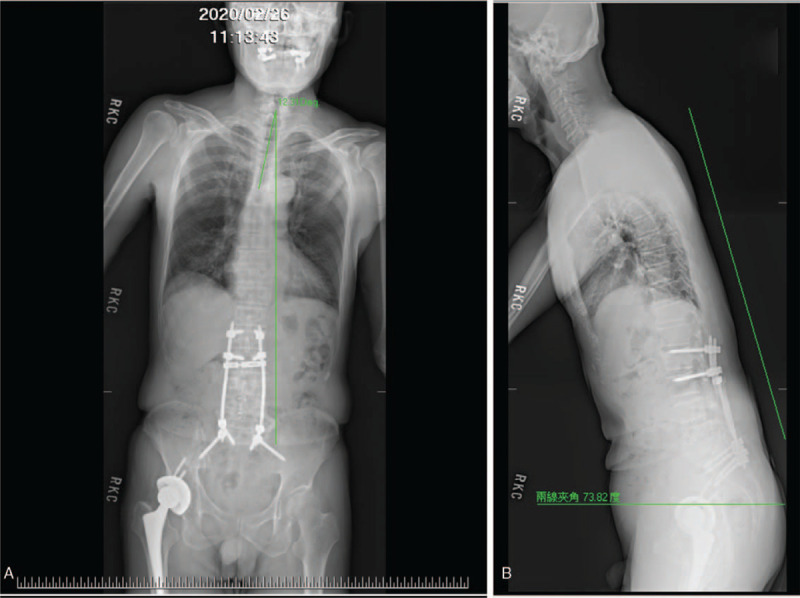
Scoliosis series and kyphosis series images of the patient. The scoliosis anteroposterior view image (2A) shows the patient's trunk flexed to the left side by 12.31 degrees; the kyphosis series lateral image (2B) shows anterior flexion by 16.18 degrees of the patient's trunk.

**Figure 3:**
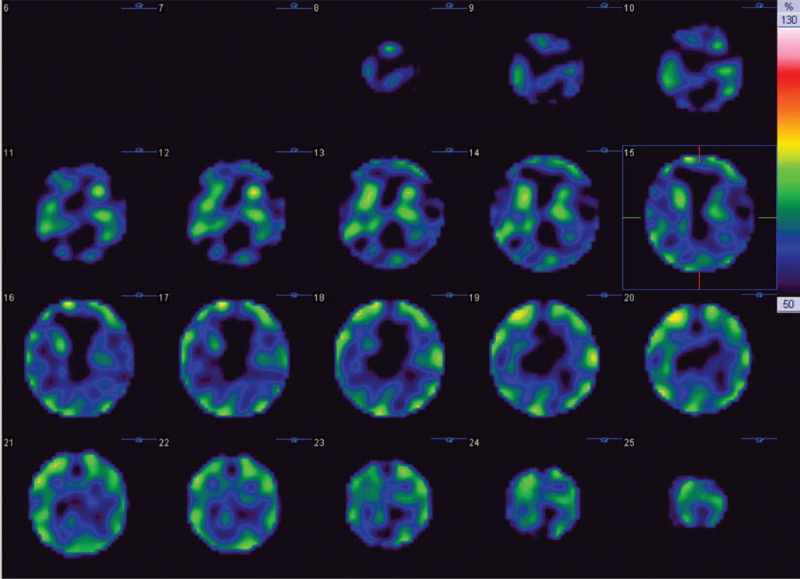
The axial technetium-99m-labeled tropanes as dopamine transporter imaging agents brain single-photon emission computed tomography scan of the patient. The images show marked reduction in the expression of dopamine transporter of the putamen and caudate nuclei of both sides. The qualitative visual scale of striatal uptake was 4. Tc-99m TRODAT-1 = technetium-99m-labeled tropanes as dopamine transporter imaging agents, SPECT = single-photon emission computed tomography.

**Figure 4:**
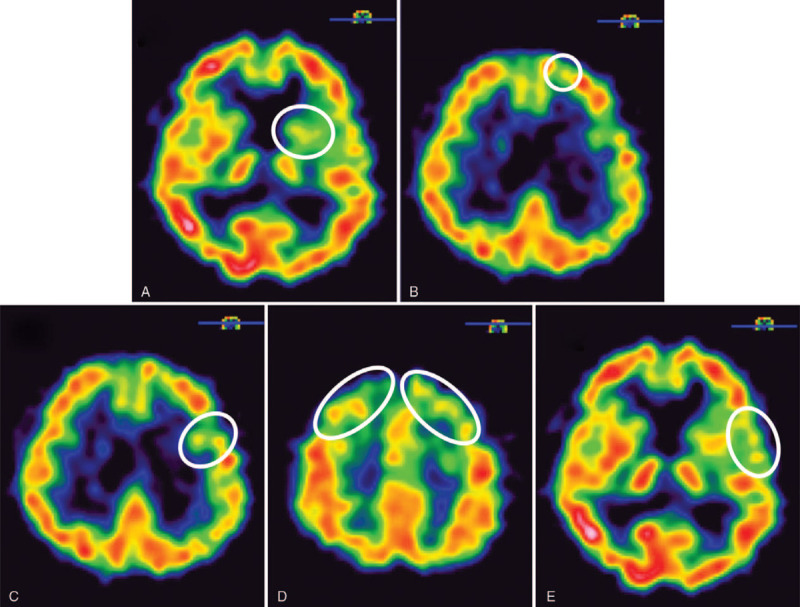
Axial Tc-99m ethylene cysteine diethyl ester brain single-photon emission computed tomography scan of the patient (Transverse view). The images reveal uneven cerebral perfusion with relative hypoperfusion (white circles) in the left parieto-occipital (4A), left frontal (4B), left parietal (4C), bilateral posterior frontal (4D), and bilateral, especially left, temporal (4E) regions. ECD = ethylene cysteine diethyl ester, SPECT = single-photon emission computed tomography.

**Figure 5:**
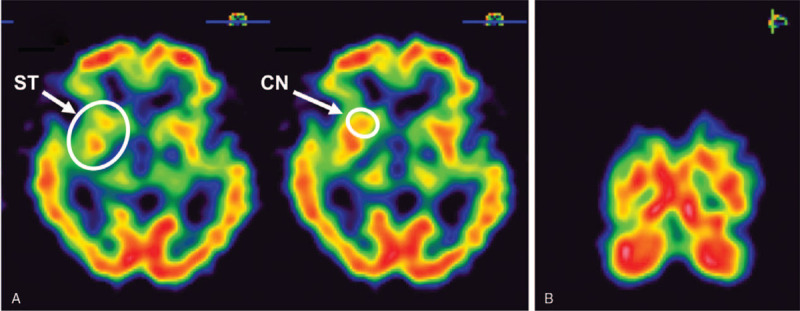
Axial Tc-99m ethylene cysteine diethyl ester brain single-photon emission computed tomography scan of the patient. 5A (Transverse view): Compared to the left side, the images show rather reduced perfusion in the right striatum that the perfusion of caudate nucleus is also severely decreased. 5B (Coronary view): The scan showed symmetric perfusion in the cerebellum of the patient. ECD = ethylene cysteine diethyl ester, SPECT = single-photon emission computed tomography.

In the OPD, the physical examination revealed the patient's trunk flexed to the left side while standing (Fig. 1); the symptom almost completely alleviated when the patient lay down in the supine position. He was unable to walk straight for more than 3 steps without any assistance. The palpitation showed tightness in the patient's left-side gluteus medius muscle and iliocostalis muscles (iliocostalis thoracis muscle, iliocostalis lumborum muscle, and iliocostalis longissimus muscle). Scoliosis series and kyphosis series X-ray were taken to survey the patient's structural deformities. The scoliosis series anteroposterior view image (Fig. 2A) showed the patient's trunk flexed to the left side by 12.31 degrees; the kyphosis series lateral view image (Fig. 2B) suggested the patient flexed anteriorly by 16.18 degrees. Based on symptoms, alleviating factors, and radiological findings, the patient was diagnosed with PS in accordance with the definition proposed by Doherty et al^[1,4]^ Tc-99m brain TRODAT-1 single-photon emission computed tomography (SPECT) and regional cerebral perfusion Tc-99m ethyl cysteinate dimer (ECD) SPECT were also arranged. TRODAT-1 SPECT scan revealed moderate to severe deficiency in the dopamine transporter uptake in the right-side putamen and the caudate nuclei of both sides (Fig. 3); ECD SPECT scan showed relative hypoperfusion in the left parieto-occipital, left frontal, left parietal, bilateral posterior frontal, and bilateral, especially left, temporal regions (Fig. 4A to 4E). Furthermore, it disclosed rather reduced perfusion in the right striatum, in which the perfusion of the right caudate nucleus (CN) was even more diminished (Fig. 5A). However, the scan image demonstrated symmetric perfusion in the patient's cerebellum (Fig. 5B). There was no marked finding in the laboratory data. Because of personal reasons, the patient refused our recommendation of admission for pharmaceutical management and was only willing to accept physical training programs at our OPD. To date, the patient has shown no sign of deterioration.

## 3 Discussion

To date, there has been no consensus on the diagnostic criteria or sign of PS, especially the degree of lateral trunk flexion required.^[4]^ In 2011, Doherty et al^[1]^ defined PS as an obvious lateral flexion of trunk greater than 10 degrees when the patient stands up, which the involuntary flexion symptoms resolve in supine position or by passive mobilization. Our patient was diagnosed with PS based on this definition.

PS can be classified into 2 patterns based on the laterality of muscle hyperactivity, compared with the side the patient's body leans to.^[5]^ Pattern I is referred to the PS of which the hyperactivity of lumbar paraspinal muscles of the patient is ipsilateral to the trunk leaning side.^[5]^ It can be further divided into 2 subtypes, namely subpattern I-I (pattern I with ipsilateral paraspinal thoracic muscle activity) and subpattern I-II (pattern I with contralateral paraspinal thoracic muscle activity).^[5]^ Pattern II is characterized by the hyperactivity of paraspinal muscles contralateral to the trunk leaning side.^[5]^ In accordance with the image results and clinical symptoms, the PS of this case is named to be pattern I as the patient's upper trunk leans to the side where the hyperactivity of lumbar paraspinal muscles is presented.

Whereas TRODAT SPECT scan has been widely adopted as a tool to diagnose and evaluate PD, ECD SPECT scan is not commonly used to assess the condition of PD. The image results of TRODAT-1 SPECT of patients with PD usually reveal reduction in dopamine uptake in striatum. In addition to the decreased striatal binding with TRODAT-1, revealed by the TRODAT scan, we find other abnormal results through ECD SPECT images, suggesting deficiency in perfusion of some regions of the brain may also contribute to the manifestation of the symptoms. Moreover, the ECD SPECT image of our patient shows symmetric isoperfusion in the cerebellum, indicating the cerebellum may not be involved with the pathophysiology of PS.

When TRODAT SPECT is used to assess the severity of PD and/or PS, the interpretation of the deficient level of dopamine transporter uptake based on the image is essential. A visualized scale, designed by Huang et al,^[6]^ to determine qualitatively the visual inspection of striatal uptake was applied in this case. Figure 3 shows severe deficiency in the dopamine transporter uptake in the left putamen of our patient. According to the assessment of the attending nuclear medicine physician from our hospital, the patient's visual scale of striatal uptake was 4, suggesting there was less than 50% and no dopamine uptake in the CN and the putamen respectively. However, as few studies have looked into the correlation between PS and image findings of TRODAT SPECT scan, we are not yet able to definitely interpret the condition of PS in this case.

In spite of previous studies that have postulated the possible pathophysiology of PS, the exact mechanism is not defined.^[2,7]^ The TRODAT SPECT (Fig. 3) and ECD SPECT (Figs. 4 and 5) images of our case show deficient function of the basal ganglia network, which is composed of multiple anatomical structures, encompassing putamen and CN. TRODAT SPECT (Fig. 3) suggests moderately to severely diminished uptake of dopamine transporter in the right putamen and bilateral caudate nuclei, while the ECD SPECT (Fig. 4) indicates decreased perfusion in the right striatum, especially hypoperfusion in the right CN. Since the CN affects locomotion and posture through striatal cholinergic interneurons, which are under dopaminergic control, we hypothesize if the basal ganglia are impaired due to PD, they can also contribute to reduced blood perfusion in the basal ganglia network, which may explain hypoperfusion in the SPECT brain images and the deficient function of basal ganglia-brain stem system of our case.

Different theories regarding possible pathophysiology of PS can be categorized into 2 groups, “central hypothesis” (the asymmetric function of the basal ganglion leads to hyperactivity of muscles for postural maintenance) and “peripheral hypothesis” (musculoskeletal problems cause postural deformity).^[1,2]^ However, Castrioto et al^[8]^ reported vast literature and data are in favor of the central hypothesis, which advocates the effect of asymmetrically functioning basal ganglion on the pathogenesis of PS. They also suggested basal ganglion with asymmetric function causes not only unequal postural muscle tone, but also unusual integration of sensorimotor information.^[8]^ Because of the misperception of orientation, patients with PS may eventually result in chronic unbalanced posture. Huh et al^[9]^ even showed most patients with PS scarcely encounter musculoskeletal deficits, indicating that the central nerve system plays a more pivotal role in the pathophysiology of PS.

Artusi et al^[2]^ demonstrated PS in patients with PD is associated with particular cognitive dysfunction. They reported PD patients with PS perform more poorly, compared with PD patients without PS, in the visual-spatial domains. The study also discovered patients with PS have a tendency for positional misperception, possibly derived from the deficit in the visual-spatial abilities.^[2]^ Moreover, while the development of spatial abilities is generally believed to be attributed to the right hemisphere of the brain, Seydell-Greenwald et al^[10]^ postulated bilateral parietal cortices participate in the formation of complex visual-spatial functions. Though unbalanced function of the basal ganglia network and impaired visual-spatial functions including parietal cortices in PS is known, very few previous studies have presented functional neuroimaging evidence to support their observation. Figure 4 demonstrates relatively diminished perfusion in the left parieto-occipital (Fig. 4A) and left parietal regions (Fig. 4C) on the ECD SPECT. Thus, we hypothesize in addition to the basal ganglia-brain stem system, other dysfunction in brain lobes also contributes to the presentation of PS in patients with PD. The aforementioned deficits in the parietal cortex may also play a role in the pathophysiology in the PS.

## 4 Conclusions

Though ECD SPECT scan is rarely used as a tool to diagnose or evaluate PS, the images still have their value. To our knowledge, our case is the first report to adopt a brain perfusion SPECT scan to present the PS in a PD patient and to further demonstrate the possible pathophysiology of PS. We believe PS in PD results from unbalanced function of the basal ganglia network and impaired visual-spatial abilities of parietal cortices bilaterally, and the cerebellum may not participate in the pathophysiology of PS due to symmetric isoperfusion in the cerebellum.

## Acknowledgment

We would like to thank the patient for his active participation in publishing this case report.

## Author contributions

**Writing – original draft:** Cheng-Jui Jamie Hung.

**Writing – review & editing:** Su-Chen Wang, Yuan-Yang Cheng, Shin-Tsu Chang.

## Abbreviations

Abbreviations: CN = caudate nucleus, ECD = ethyl cysteinate dimer, OPD = outpatient department, PD = Parkinson disease, PS = Pisa syndrome, SPECT = single-photon emission computed tomography.
